# Investigating the Relationship Between Mitral Leaflet Separation Index and Severity of Mitral Valve Stenosis Using Two and Three-Dimensional Measurement Methods in Trans Esophageal Echocardiography

**DOI:** 10.34172/jcvtr.33359

**Published:** 2026-06-30

**Authors:** Alaaldin Hoshmand, Naser Khezerlouy-Aghdam, Rezvanieh Salehi, Somayeh Abolhasani, Elnaz Javanshir

**Affiliations:** ^1^Cardiovascular Research Center, Tabriz University of Medical Sciences, Tabriz, Iran; ^2^Department of Biochemistry, Faculty of Medicine, Urmia University of Medical Sciences, Urmia, Iran

**Keywords:** Echocardiography, Mitral leaflet separation, Mitral stenosis, Planimetry

## Abstract

**Introduction:**

Cardiologists use several echocardiographic methods to evaluate the Mitral Stenosis (MS) severity. Determination of mitral valve area (MVA) by three- dimensional transesophageal echocardiography (3D TEE) planimetry has the best correlation with anatomic area of the mitral orifice and is probably the reference method. This study was designed to examine the relationship between mitral leaflet separation index (MLSI) measured by TEE which is a simple and accessible method and MVA calculated by 3D Multiplanar reconstruction (3D-MPR) planimetry in TEE as the reference but less accessible method for evaluation the MLSI accuracy in prediction of the severity of MS.

**Methods:**

72 patients with MS were included in this study. Exclusion criteria were suboptimal echocardiographic images, severe mitral valve calcification, artificial heart valves, a history of mitral valve repair surgery, or contraindications to TEE. After obtaining written informed consents from all patients. Comprehensive TTE and TEE were performed and MLSI by 2D TTE and 2D TEE and MVA by 3D MPR planimetry were assessed. SPSS version 25 was used for performing the analysis and a *P*-value less than 0.05 was considered statistically significant.

**Results:**

The average age of the patients was 57.81±12.22 and of the patients were female. There was a significant correlation between MVA by 3D MPR and MLSI 2D TTE (r=0.94, *P*<0.01) and MLSI 2D TEE (r=0.96, *P*<0.01) methods.

**Conclusion:**

MLSI 2D TTE and MLSI 2D TEE are positively correlated to MVA measured by 3D MPR planimetry and MLSI can be useful in discriminating different grades of MS severity.

## Introduction

 Mitral stenosis (MS) is a form of valvular heart disease which is characterized by narrowing of the mitral valve orifice. Symptoms of MS include dyspnea, orthopnea and paroxysmal nocturnal dyspnea followed by palpitations and chest pain.^1^ The most common etiology of MS is rheumatic fever (RF). Which has an incidence of 1 in 100,000 in developed countries.^2^ However, developing nations have a higher prevalence of RF.^1^ MS is evaluated using various methods like electrocardiogram (ECG), chest X-ray and echocardiography with parameters such as pressure half time (PHT), mean Doppler gradient, pulmonary pressures and determination of mitral valve orifice area (MVA) by planimetry.^3-7^ Three-dimensional transesophageal echocardiography (3D TEE) is considered the most precise approach for estimating the valve area. However, due to its limited accessibility, this method is not commonly employed in routine clinical practice.^8-10^ The mitral leaflet separation index (MLSI) measures the average distance between the free edge of the mitral leaflets in orthogonal views.^11^ MLSI was recently introduced as a dependable supporting parameter for determination MS severity.^12^ Bigdelu et al. claim that calculation of MLSI through two-dimensional transthoracic echocardiography (TTE) is highly effective in assessing the severity of MS and is closely associated with the results obtained from three-dimensional transesophageal echocardiography (3D TEE) planimetry.^13^ This study will examine the relationship between MLSI measured in TTE (Parasternal long axis and apical 4 chamber views) and in TEE (Midesophageal, four chamber 0’ and long axis 120 ‘views) and MVA calculated by 3D-MPR Planimetry method in TEE in determining the severity of mitral valve stenosis.

## Materials and Methods

###  Population

 72 patients with MS, getting their diagnosis by TTE, who were referred to the echocardiography unit of Shahid Madani Hospital, Tabriz, Iran in order to evaluation of MS severity using TEE were entered in this study. Exclusion criteria were patients with suboptimal echocardiographic images, severe mitral valve calcification, artificial heart valves, a history of mitral valve repair surgery, or contraindications to TEE.

###  Echocardiographic Image Acquisition

 Written informed consents were obtained from all patients before undergoing echocardiography. After performing comprehensive TTE using a commercial ultrasound system (Philips EPIQ 7C ultrasound machine), MLSI 2D TTE was obtained from the mentioned views with the same rules as mentioned in TEE. Then TEE was performed using a matrix-array transducer under conscious sedation. MLSI 2D TEE was calculated by averaging the maximal distance between the tips of the leaflets at diastole in apical four chamber and long axis views (Figure 1). The average numbers of these two views were calculated to obtain the MLSI. Measurements were averaged for three consecutive cardiac cycles in patients with normal sinus rhythm, and five beats in those with atrial fibrillation (AF). All patients were received propofol with a dose of less than 1 mg/kg for sedation before starting the echocardiography. Also, continues cardiac monitoring and pulse oximetry was performed for all the patients during the echocardiography.

**Figure 1:**
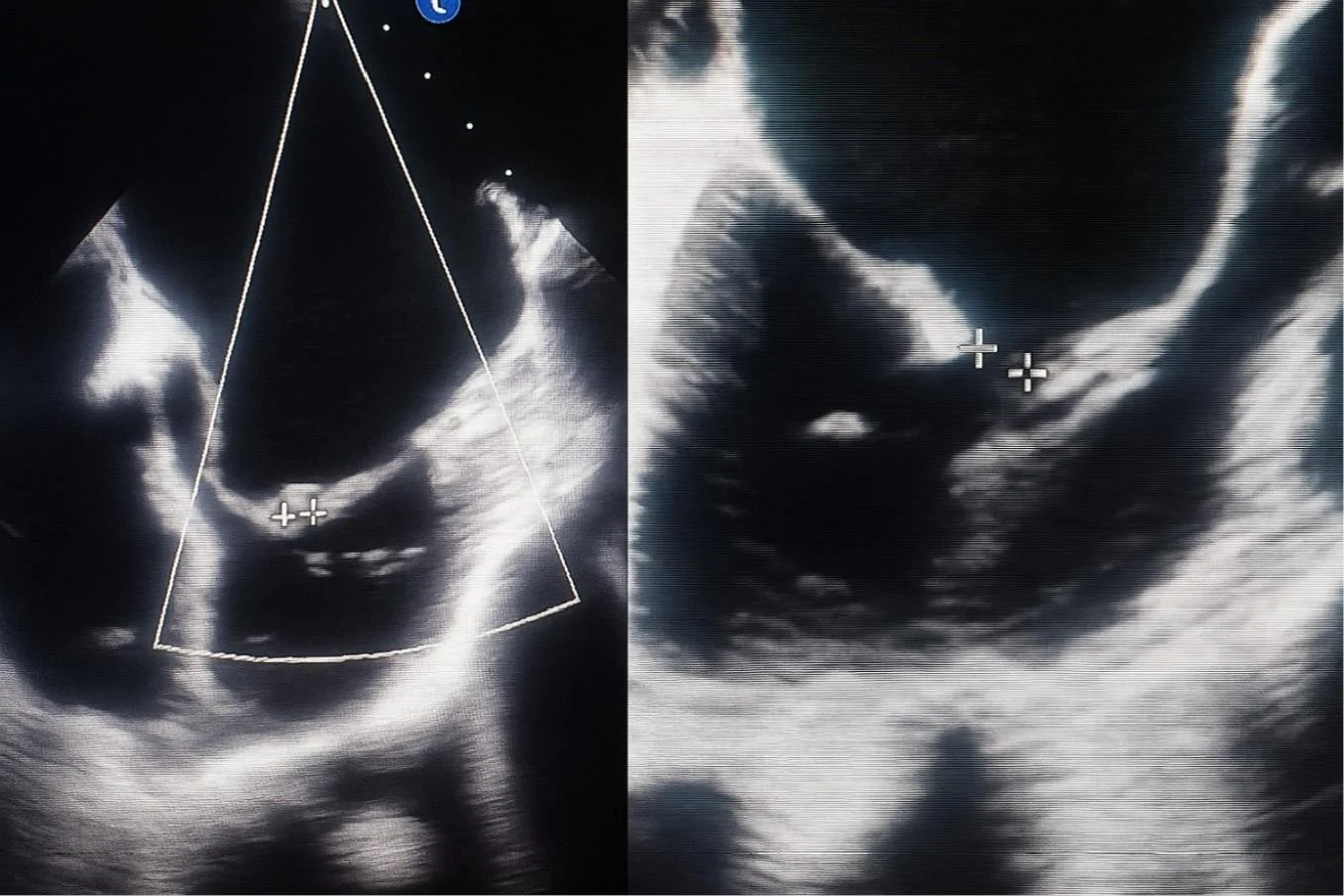
Measurement of MLSI using 2D TEE

 After obtaining the appropriate bi-commissural view, a three-dimensional view of the mitral valve surface was obtained using 3D zoom and the images were saved. Digitally saved images were reviewed offline, a suitable three-dimensional view of the mitral valve was obtained and the valve area (MVA) was calculated using QLAB software. MVA planimetry was assessed after obtaining an orthogonal plane through the narrowest orifice of the mitral valve in diastole (Figure 2). Based on the obtained values, the severity of MS was divided into categories of very severe MS (MVA ≤ 1mm^2^), severe MS (MVA ≤ 1.5mm^2^) and progressive MS (MVA > 1.5mm^2^).

**Figure 2:**
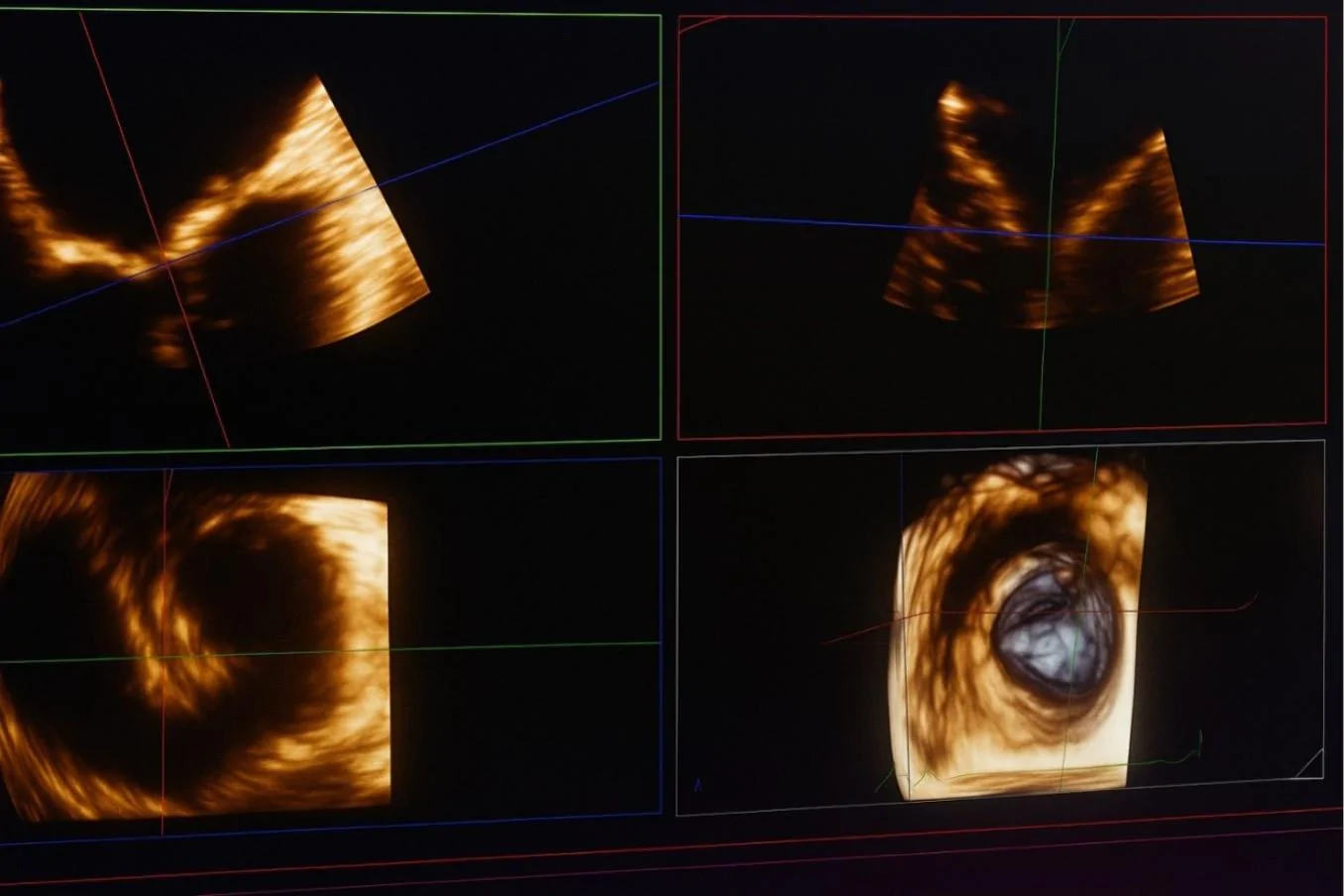
MVA planimetry using 3D TEE multiplaner reconstruction

 Finally, the obtained 2D TTE and 2D TEE MLSI values were compared with 3D MVA and the MLSI values were determined in different degrees of MS based on 3D MVA.

###  Statistical Analysis

 Data acquisition and statistical processing were carried out using SPSS version 25.0. Continuous variables were expressed as mean ± standard deviation (SD. (Categorical data were presented as absolute numbers (percentage). Normality of MLSI values was checked using Kolmogorov Smirnov test. The MLSI 2D TTE and TEE for progressive, severe, and very severe MS was analyzed using analysis of variance to determine whether the index could discriminate between different categories of MS severity. The MLSI 2D was further correlated with MVA planimetered by 3D MPR using linear regression analysis. Analyses were performed overall and in subgroups of patients according to the rhythm and the presence of MR. Receiver operating characteristics curve (ROC) was used to obtain the best MLSI 2D cut-off values for different grades of MS. Regression analysis were performed in the first group to derive a regression equation of MVA3D against MLSI2D. The MVA was calculated in the second group by this equation and compared with MVA3D, using paired t-test. Intra- and inter-observer reproducibility and the agreement between different methods of MVA estimation were evaluated using intra-class correlation coefficients (ICC). *P*-values < 0.05 were considered of statistical significance.

## Results

 72 patients aged 19 to 82 were investigated in this study. Demographic data and risk factors of these patients are shown in Table 1. The average age of the patients was 57.81 ± 12.22. 48 (66.7%) patients were female and 24 (33.3%) were male. 16 (22.2%) patients had a history of percutaneous trans mitral commissurotomy (PTMC). Also, 29 (40.8%) patients had a history of hypertension (HTN). 38 (52.8%) patients had functional class II (FCII) dyspnea on exertion (DOE). 38 (52.8%) patients had sinus rhythm and 34 (47.9%) patients had atrial fibrillation (AF) rhythm. 12 (16.7%) patients had diabetes Mellitus (DM), 11 (15.3%) patients had ischemic heart disease, and 5 (6.9%) patients underwent percutaneous coronary intervention (PCI). No patient had a history of prior coronary artery bypass graft surgery (CABG) and 12 (18.2%) patients had cerebrovascular disease. Table 2 demonstrates TTE data of patients. Mean left ventricular ejection fraction (LVEF) was 49.78 ± 7.58 with the lowest value of 25% and the highest value of 60%. The average LV end diastolic diameter (LVEDD) was also reported 4.71 ± 0.56 cm with the lowest value being 3.4 cm and the highest value being 7.2 cm. Also, the average LV end systolic diameter (LVESD) was reported as 3 ± 0.53 cm with the lowest amount being 2.2 cm and the highest amount being 5.1 cm. At least moderate aortic insufficiency (AI) was recognized in 35 (48.6%) of patients. 9 (12.5%) patients had at least moderate aortic stenosis (AS). And 50 (69.4%) patients showed at least moderate tricuspid regurgitation (TR), and at least moderate mitral regurgitation (MR) in 53 (73.6%) were diagnosed. Pulmonary stenosis (PS) was not observed in any patients. MS severity was reported progressive in 15 (20.8%) patients, severe in 24 (33.3%) patients and very severe in 33 (45.8%) patients. One-way analysis of variance was used to investigate the ability of MLSI 2D TTE and MLSI 2D TEE methods in differentiation of different degrees of MS compared to MVA 3D MPR planimetry method. It was observed that both methods correctly discriminate between very severe and progressive MS (*P*-value < 0.01). The average MLSI 2D TTE was reported 12.61 ± 1.16 mm in progressive MS patients, 9.39 ± 0.91 mm in severe MS patients, and 7.69 ± 1.09 mm in very severe MS patients. The average MLSI 2D TEE was reported as 12.64 ± 1.07 mm in progressive MS patients, 9.61 ± 1.04 mm in severe MS patients, and 7.61 ± 1.14 mm in very severe MS patients. Moreover, average MVA 3D MPR planimetry in progressive MS patients was the highest at 1.81 ± 0.18 cm^2^, followed by 1.31 ± 0.18 cm^2^ in patients with severe MS and finally 0.86 ± 0.16 cm2 in patients with very severe MS. Tukey’s post hoc test showed this difference between all groups statistically significant. These results are presented in Table 3. Correlation between the values of MLSI 2D TTE, MLSI 2D TEE and MVA 3D MPR planimetry were checked by Pearson correlation coefficient. It was observed that there is a significant correlation between MVA 3D planimetry with both MLSI 2D TTE (r = 0.94, *P* < 0.001) and MLSI 2D TEE (r = 0.96, *P* < 0.001) methods (Figures 3 and 4). Linear regression was used to investigate the correlation effect between MVA 3D planimetry, MLSI 2D TEE and MLSI 2D TTE. Results showed that the regression coefficient for MLSI 2D TEE was 0.96 and it was 0.92 for MLSI 2D TTE in all patients including patients with AF rhythm and patients with MR (Figures 3 and 5). Roc curve was used in order to detect the severity of MS by MLSI 2D TEE and MLSI 2D TTE method. Results showed that the cut point value of MLSI 2D TEE in diagnosis of very severe MS was ≤ 8.62 mm with a sensitivity of 94% and a specificity of 82% and an area under cover (AUC) value of 95%. Also, the cut point value for diagnosing patients with progressive MS was ≥ 10.82 mm with 100% sensitivity and 99% specificity and AUC value of 98% (Figures 4 and 6). Patients with severe MS were reported between 8.63 and 10.81 mm. Moreover, the cut point value of MLSI 2D TTE for very severe MS was ≤ 8.57 mm with 92% sensitivity and 82% specificity and 93% AUC value. Also, the cut point value for progressive MS was ≥ 10.85 mm with 100% sensitivity and 99% specificity and AUC value of 99% (Figures 4 and 6).

**Table 1:** Demographic data and risk factors of patients

**Characteristic **		
Age		57.81[12.22]
Gender	Male	24 (33.3)
	Female	48 (66.7)
PTMC history		16 (22.2)
HTN		29 (40.8)
DOE (FC)	I	2 (2.8)
	II	31 (43.7)
	III	27 (38.0)
	IV	11 (15.5)
Sinus Rhythm		38 (52.8)
AF		34 (47.9)
DM		12 (17.9)
Ischemic Heart Disease		11 (16.4)
Cerebrovascular disease		12 (18.2)

The numeric data are presented in median [IQR] or mean ± SD, based on the normality of distributions and nominal data are presented in number (percentage). PTMC: Percutaneous Trans Mitral Commissurotomy, HTN: Hypertension, DOE: Dyspnea On Exertion, FC: Function Class, AF: Atrial Fibrillation, DM: Diabetes Mellitus, PCI: Percutaneous coronary Intervention, CABG: Coronary Artery Bypass Graft.

**Table 2:** Echocardiography data of patients

**Echocardiography characteristic**		
AI ≥ Moderate		35 (48.6)
AS ≥ Moderate		9 (12.5)
TR ≥ Moderate		50 (69.4)
MR ≥ Moderate		53 (73.6)
MS	Progressive	15 (20.8)
	Severe	24 (33.3)
	Very Severe	33 (45.8)
LVEF (%)		49.78 [7.58]
LVEDD (cm)		4.71 [0.56]
LVESD (cm)		3.09 [0.53]
SPAP (mmHg)		41.58 [15.58]
Trans-Mitral Gradiant (mmHg)		7.91 [3.82]
MLSI by 2D TEE (mm)		9.32 [2.21]
MLSI by 2D TTE (mm)		9.28 [2.14]

The numeric data are presented in median [IQR] or mean ± SD, based on the normality of distributions and nominal data are presented in number (percentage). AI: Aortic Insufficiency, AS: Aortic Stenosis, TR: Tricuspid Regurgitation, MR: Mitral Regurgitation, MS: Mitral Stenosis, PR: Pulmonary Regurgitation, LVEF: Left Ventricular Ejection Fraction, LVEDD:LV- End Diastolic Diameter, LVESD:LV- End Systolic Diameter, SPAP: Systolic Pulmonary Artery Pressure, MLSI: Mitral Leaflet Separation Index, 2D: Two Dimensional, TEE: Trans-esophageal Echocardiogram, TTE: Trans-thoracic echocardiogram.

**Table 3:** Comparison of three methods MLSI 2D TTE, MLSI 2D TEE and MVA 3D planimetry in different degrees of mitral valve stenosis

**Characteristic**	**Progressive**	**Severe**	**Very Severe**	***P*** **-value**
MVA3D planimetry (cm^2^)	1.81 ± 0.18	1.31 ± 0.18	0.86 ± 0.16	<0.01*
MLSI2D TTE (mm)	12.61 ± 1.16	9.39 ± 0.91	7.69 ± 1.09	<0.01*
MLSI2D TEE (mm)	12.64 ± 1.07	9.61 ± 1.04	7.61 ± 1.14	<0.01*

**Figure 3:**
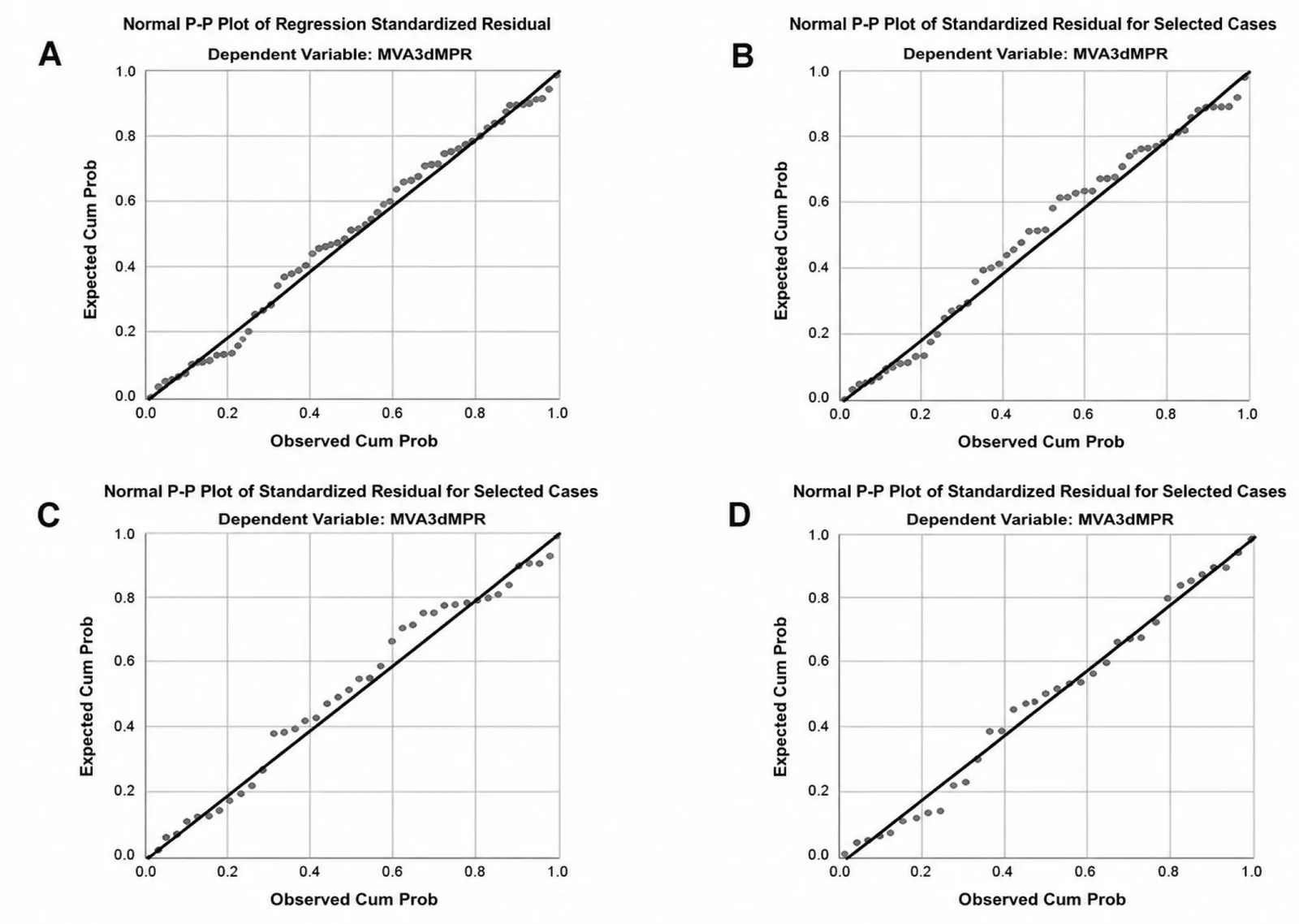
Regression coefficient between MVA 3D MPR planimetry and MLSI 2D TEE for all patients (A), patients with ≥ moderate MR (B), patients with sinus rhythm (C) and patients with AF rhythm (D)

**Figure 4:**
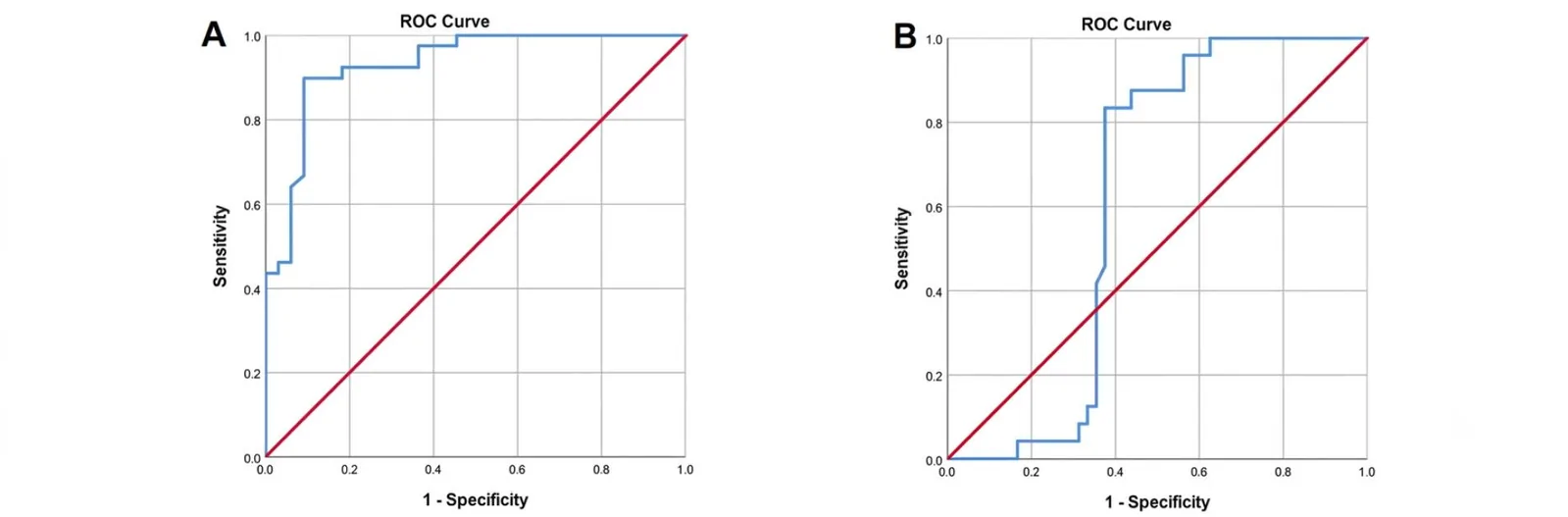
MLSI 2D TEE Roc curve in patients with very severe MS (A) and patients with progressive MS (B)

**Figure 5:**
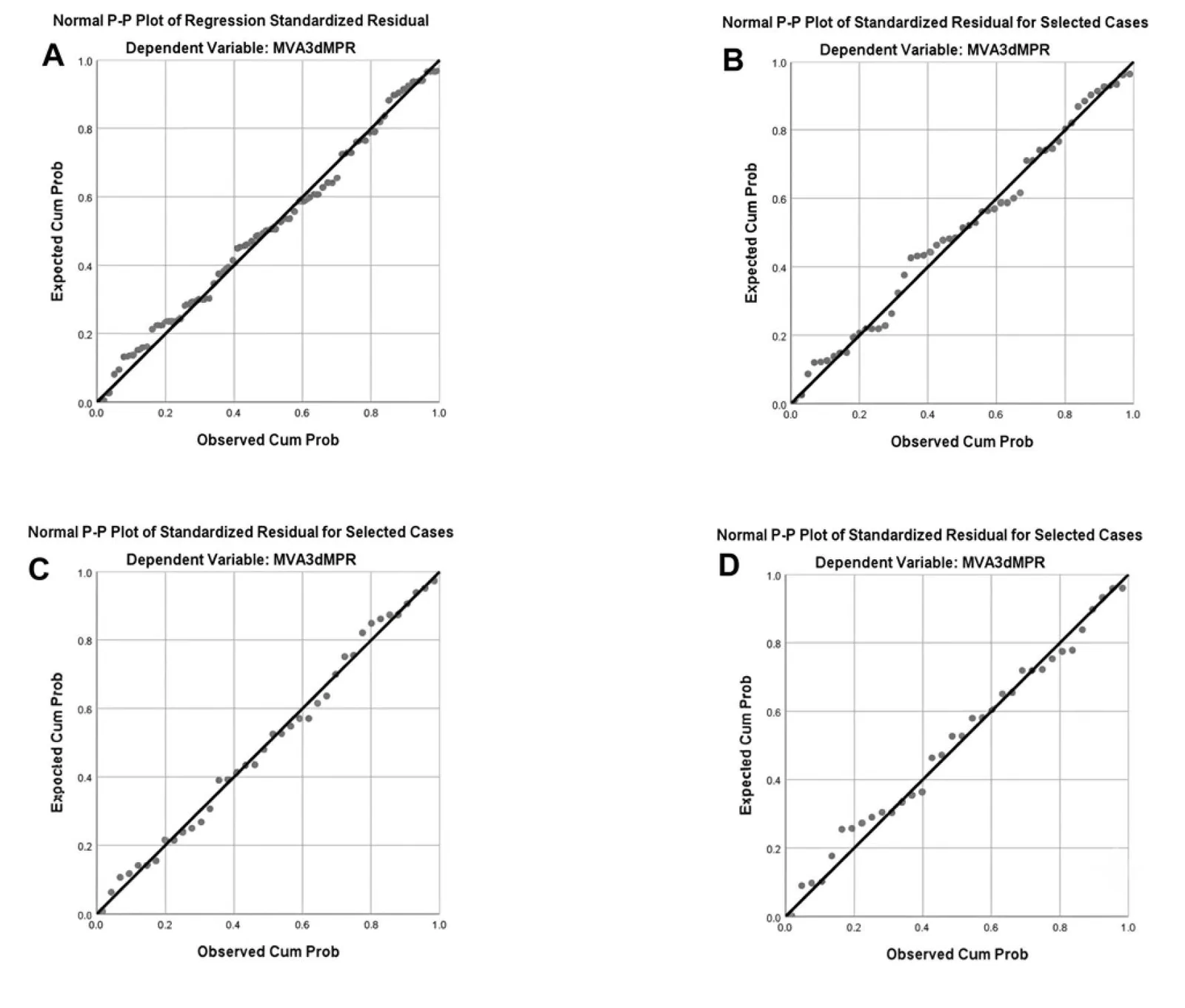
Regression coefficient between MVA 3D MPR planimetry and MLSI 2D TTE for all patients (A), patients with ≥ moderate MR (B), patients with sinus rhythm (C) and patients with AF rhythm (D)

**Figure 6:**
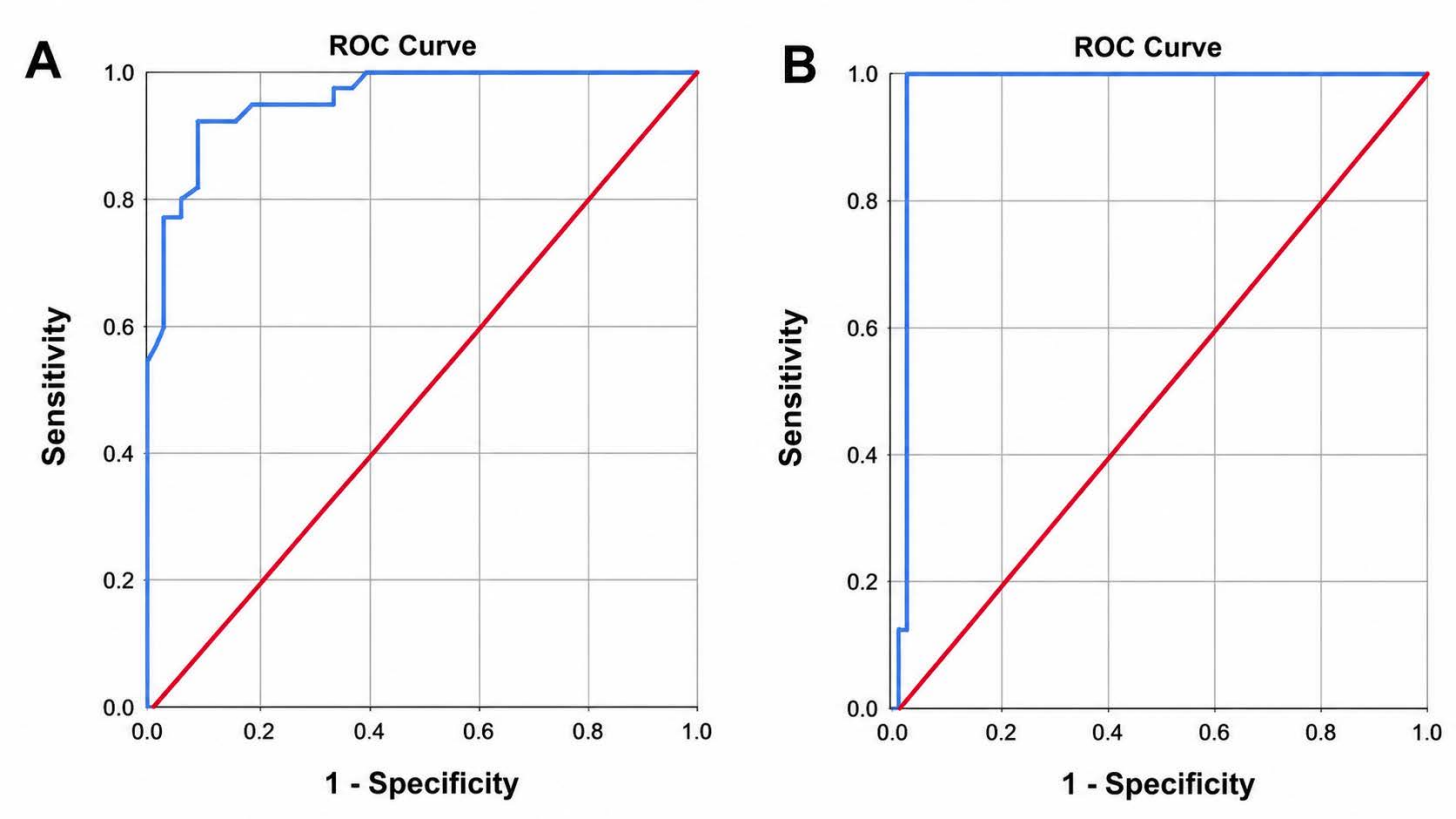
MLSI 2D TTE Roc curve in patients with very severe MS (A) and patients with progressive MS (B)

## Discussion

 In this study relationship between MLSI and severity of MS was investigated using 2D and 3D measurement methods in TEE. Study results claim that MLSI measured by 2D TTE and 2D TEE correlates well with MVA measured by 3D MPR planimetry. Although echocardiography is recommended as the most accurate approach for diagnosis of MS and evaluation of its progression, a gold standard for the assessment of MS severity on TTE is yet to be introduced.^14,15^ There are several echocardiographic methods used by cardiologists to evaluate the MVA. However, each technique has a specific potential limitation. For example, the duration of pressure half-time (PHT) relies heavily on hemodynamic factors and can be influenced by alterations in preload and left ventricular compliance. The trans-mitral gradient and continuity equation are also notably impacted by transvalvular flow, cardiac output, and the concurrent presence of mitral regurgitation (MR).^16,17^ Guidelines suggest that the MS severity should rather be assessed by a multimodality approach that determines valve areas, mean Doppler gradients, and pulmonary pressures. Therefore, 3D MPR planimetry is reported to have the best correlation with anatomic MVA. However, it can become highly operator-dependent and laborious due to variable geometry of stenotic mitral valve orifice, precise plane orientation at the tip of the leaflets and measuring the narrowest part of the mitral funnel.^9,18^ In a study by Rafaat et al. TTE MLSI 2D was claimed to act as a reliable measure of MS severity in the majority of patients with MS. They also suggest that MLSI is a technically easier method specially for less-experienced echocardiographers.^11^ In this study it was the same as 2D TEE MLSI and 2D TTE MLSI techniques were found to be correctly discriminating between stages of MS. Studies claim that measuring the MLSI on 2D imaging is a technically easier compared to planimetry and also accurate technique as the narrowest part of the mitral funnel can be easily identified.^19^ MLSI also has a very good correlation with MVA2D TTE planimetry and is very easy to obtain in the hands of the inexperienced and in special setting like Cath lab.^19-21^ Recent studies also confirm this as Seow et al. found a correlation between MLSI and MVA by planimetry and PHT using 2DTTE.^19^ Thomas et al.also reported a relation between MLSI and MVA by planimetry and PHT.^22^ Finally, in a recent research by Bigdelu et al, MLSI 2D TTE also showed an excellent ability to assess MS severity and it correlated well with planimetered MVA measured by 3D MPR.^13^ The results of our study were similar to all previous research regarding the ability of MLSI 2D in assessing MS severity. However this study had small difference with Bigdelu et al. study regarding the calculated amounts of 2D TTE MLSI. Possible explanations could be the interobserver variability as well as this fact that unlike Bigdelus study patients with a history of PTMC were included in this study. To our knowledge this is the first study that performed TEE besides TTE for getting MLSI as well as the use of 3D TEE as a comparator. This was the main strength of this study. However, this study has some limitations. The population of this study was statistically acceptable, but due to the fact that people were evaluated in three different subgroups, the number of people in the progressive group was less than the other two groups. By choosing a larger statistical population, the number of people in different subgroups will be more and ultimately more reliable statistical results will be obtained.

## Conclusion

 This study found that the MLSI measured by 2D TTE and 2D TEE are significantly correlated with MVA obtained by 3D MPR planimetry and it can be very useful in discriminating different grades of MS severity.

## Competing Interests

 The authors report no relationships that could be construed as a conflict of interest.

## Ethical Approval

 The ethics committee of the Tabriz University of Medical Science reviewed and approved the study protocol (Ethical Code: IR.TBZMED.VCR.REC.1403.162).
